# Embedding weight management into safety-net pediatric primary care: randomized controlled trial

**DOI:** 10.1186/s12966-017-0639-z

**Published:** 2018-01-22

**Authors:** Judith Wylie-Rosett, Adriana E. Groisman-Perelstein, Pamela M. Diamantis, Camille C. Jimenez, Viswanathan Shankar, Beth A. Conlon, Yasmin Mossavar-Rahmani, Carmen R. Isasi, Sarah N. Martin, Mindy Ginsberg, Nirupa R. Matthan, Alice H. Lichtenstein

**Affiliations:** 1 Department of Epidemiology and Population Health, Albert Einstein College of Medicine, 1300 Morris Park Avenue, Bronx, NY 10461 USA; 2 Department of Pediatrics, Albert Einstein College of Medicine, Jacobi Medical Center, 1400 Pelham Pkwy S, Bronx, NY 10461 USA; 3 Cardiovascular Nutrition Laboratory, JM USDA Human Nutrition Research Center on Aging, Tufts University, 711 Washington St, Boston, MA 02111 USA

**Keywords:** Weight management, Family-based intervention, Safety net care

## Abstract

**Background:**

Implementing evidence-based recommendations for treating pediatric overweight and obesity is challenging in low-resource settings. We conducted a randomized controlled trial to evaluate the effects of implementing the American Academy of Pediatrics overweight/obesity recommendations using a Standard Care approach alone or with the addition of an enhanced program in a safety-net pediatric primary care setting (located in Bronx, New York, United States).

**Methods:**

In a 12-month trial, families of children (age 7–12 years; body mass index ≥85th American percentile for age and sex; 74% self-identified as Hispanic/Latino; *n* = 360) were randomly assigned to receive Standard Care Alone or Standard Care + Enhanced Program. An English/Spanish bilingual staff provided the Standard Care Alone consisting of quarterly semi-structured pediatrician visits targeting family-based behavioral changes. The Standard Care + Enhanced Program was enriched with eight Skill-Building Core and monthly Post-Core Support sessions.

**Results:**

The mean body mass index Z-score declined in both arms (*P* < 0.01) with no significant difference between the Standard Care Alone (0.12 kg [SE: 0.03]) and Standard Care + Enhanced Program (0.15 kg [SE: 0.03]) arm (*P* = 0.15). Compared to the Standard Care Alone, the Standard Care + Enhanced Program resulted in significantly greater improvements in total cholesterol (*P* = 0.05), low-density lipoprotein cholesterol (*P* = 0.04), aspartate aminotransferase (*P* = 0.02)*,* and alanine transaminase (*P* = 0.03) concentrations.

**Conclusions:**

Safety-net primary care settings can provide efficacious pediatric weight management services. Targeted family-based behavioral counseling helps overweight/obese children achieve a modest body mass index Z-score improvement. A more intensive lifestyle intervention program may improve some metabolic parameters.

**Trial registration:**

ClinicalTrials.gov Identifier: NCT00851201. Registered 23 February 2009.

**Electronic supplementary material:**

The online version of this article (10.1186/s12966-017-0639-z) contains supplementary material, which is available to authorized users.

## Background

Evidence-based care standards from the American Academy of Pediatrics for the prevention and treatment of obesity in children recommend supporting families to make lifestyle changes, addressing excess body weight in children as a chronic condition, and providing advice about weight-related health risk factors [1–4]. Quality improvement efforts have demonstrated that electronic health record (EHR) prompting can improve pediatric weight-related assessment and intervention as integral components of primary care [5]. A meta-analysis of randomized controlled trials found that intervention intensity via contact frequency of behavioral family lifestyle interventions predicted greater weight improvement (based on body mass index (BMI) z score reduction) in children [6]. Other research has demonstrated that improvement in BMI Z- score predicts improvement in cardiometabolic risk markers [7]. Despite the strong evidence that intensive family-based interventions can reduce obesity and its co-morbidities, implementation of such programs is limited especially in low income predominately minority communities [8], and attrition rates are frequently greater than 50% [9].

Surveys by the Children’s Hospital Association found that only 40% of hospitals reported having established procedures for identifying children for whom obesity was a potential health risk [10] and that low-income parents, who had dropped out of family weight management programs/clinics, indicated that transportation and inflexible program/clinic schedules were barriers to their participation [9, 11]. Other social and environmental barriers and challenges that low-income minority families face include unsafe streets, poor neighborhood recreational facilities, lack of sports options, abundance of fast food and street vending outlets, limited number and poor quality of grocery stores, long work hours, influence of extended family, and promotion of high calorie foods as inexpensive treats/rewards [8, 12–14]. However, little is known about how to integrate family weight management services that address these barriers and minimize dropout in safety-net health care settings.

Our practice-based study evaluated the efficacy of novel strategies for embedding weight management services into pediatric ambulatory care in a publically funded safety-net care system.

Drawing on the social-ecological framework, we considered motivational enhancement and cognitive behavioral strategies to develop intervention components that addressed the weight management challenges the families were likely to encounter in their home and community environment [15–19]. Healthy food and physical activity behaviors were promoted as social norms based on social marketing principles using available resources including Bronx and New York City healthy lifestyle campaigns e.g., farmer’s markets, community gardens, free parks programs and other community resource booklets/guides (See Additional file 1: Table S1 for details). Our aim was to compare the efficacy of providing a bilingual weight management intervention as a high quality Standard Care Alone delivered by two primary care pediatricians, versus providing the Standard Care + Enhanced Program, which added skills-based core modules and post-core support from a multidisciplinary team to facilitate intervention tailoring. We hypothesized that children from families randomized to the Standard Care + Enhanced Program would have greater improvements in body mass index (BMI) Z-scores and metabolic parameters than children from families randomized to the Standard Care Alone. Our Enhanced Program evaluation asked parent/guardian to rate the helpfulness of intervention components and about factors that enhanced motivation and barriers that impeded changing eating and physical activity behaviors.

## Methods

### Setting

The study was conducted in a safety-net pediatric primary care setting in Jacobi Medical Center (Bronx, New York, United States). Health services for children were predominately covered by public funding through Medicaid [20] and the Child Health Insurance Plan [21]. Jacobi Medical Center is a component of New York City’s Health and Hospital Corporation municipal health system and is affiliated with the Albert Einstein College of Medicine.

### Study participants

Inclusion criteria were age 7 to 12 years and BMI ≥85th United States CDC BMI percentile [22] for age and sex. Exclusion criteria included chronic illness (e.g., diabetes), impairments that would affect ability or safety to follow the study protocols, treatment with medications known to affect body weight, and enrollment in another weight management program within 2 years.

### Trial design

The study was a two-arm randomized, controlled, parallel-group trial comparing Standard Care Alone (quarterly pediatrician visits to address weight management recommendations) to Standard Care + Enhanced Program*.* The Enhanced Program added a behavioral change component (eight skill-building core sessions and monthly post-core support sessions focused on dietary modification and increased physical activity). Additional file 1: Table S1 provides an overview of the intervention components by randomization group.

### Protection of human subjects and recruitment, enrollment, and randomization procedures

**Fig. 1:**
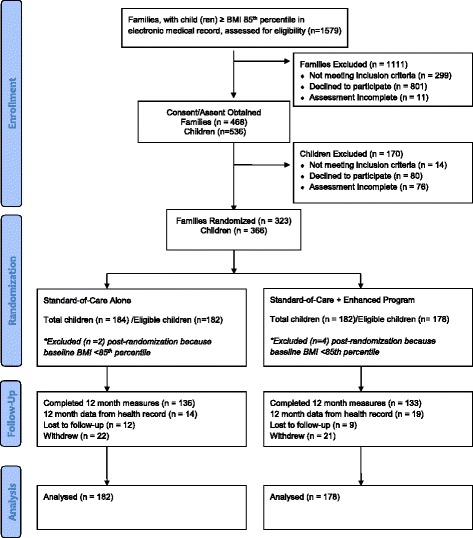
CONSORT (CONsolidated Standards Of Reporting Trials) Flow Diagram

All study procedures were approved by the Institutional Review Boards of the Albert Einstein College of Medicine and Tufts University. Recruitment and enrollment occurred from 27 July 2009 through 30 December 2011 in collaboration with primary care providers. All study materials were available in English and Spanish; the study staff communicated in the language preferred by the families. The study recruitment was designed to interface with the EHR System, which prompts primary care providers to measure height and weight at each visit and flags children with a BMI ≥ 85th percentile for primary provider review. A study assistant used the EHR flagging system to identify eligible children (*n* = 1579). Each primary care provider received a list of his or her potentially eligible patients to review, and children (*n* = 299) were ineligible based on feedback from the primary care provider. For the study pediatricians who provided the Standard Care weight management consults, patient enrollment was limited to new patients. These (embedded) pediatricians had already referred their overweight patients to the designated weight management service sessions. Therefore, their ongoing patients with a BMI ≥ 85th percentile would have received weight management services within the past 2 years, making them ineligible. After review of eligibility with primary care providers, study staff provided a brief study overview to the parent/guardian of eligible children and addressed questions. Additionally, flyers were posted in the clinic area to reinforce the recruitment efforts and stimulate self-referrals. Written consent was obtained from all parent/guardian, and assent was obtained from child(ren) entered into the study. Study staff opened sequentially numbered, opaque, sealed envelopes to randomize families based on a computer generated 1:1 allocation schedule created by the data unit. The Consolidated Standards of Reporting Trials (CONSORT) Flow Diagram (Fig. 1) and CONSORT Checklist (see Additional file 2) provide an overview of the two arm randomized, controlled, parallel-group trial phases (enrollment, intervention allocation, follow-up, and data analysis).

## Intervention methods

### Standard care

The Standard Care intervention was based on the American Academy of Pediatrics evidence-based recommendations using intervention materials selected during pilot testing [1–3]. Two bilingual primary care pediatricians, who were embedded in the practice, provided the Standard Care intervention for all of the study families.

#### Kid-WAVE introduction

To foster early engagement children were given the Kid-Weight, Activity, Variety, and Excess (WAVE) Get Healthy card game [23]. The 12-item WAVE card game includes questions adapted from the Youth Behavioral Risk Factor Survey [23–25] to help children choose behavioral targets (e.g., playing active video games, eating more vegetables, avoiding super-sizing or drinking water rather than sugar-sweetened beverages).

#### Standard care pediatrician visits

The pediatrician visits were provided quarterly in designated clinical sessions reserved for the weight management study patients. Case discussions and observations were used to establish and maintain intervention standardization and fidelity. The Standard Care pediatrician visit procedures were the same for both of the treatment arms.

The initial visit was a comprehensive, structured 40-min appointment to assess weight-related issues and to engage both the child(ren) and parent(s)/guardian(s) in developing intervention goals collaboratively. The pediatricians used the 35-item Pediatric Symptom Checklist to screen for emotional and behavioral dysfunctions [26, 27]; consistent with practice guidelines, scores ≥28 served as a basis for a social worker referral [26, 27]. The 5-item Habits questionnaire was used to assess dietary, physical activity and sedentary behaviors [28]. The results were used for making referrals to the registered dietitian and guiding the pediatricians’ use of New York City Department of Health and Mental Hygiene weight management tools (Additional file 3: Table S2). The Habits questionnaire addressed meals (e.g., eating as a family and avoid eating while watching TV), fruit and vegetable intake (e.g., increasing serving, excluding juices), beverage intake (e.g., decreasing sugar-sweetened beverages, choosing 1% fat milk and water), fast food (e.g., decreasing frequency, avoiding super-sizing and choosing healthier options), and physical activity/sedentary behavior (e.g., increasing moderate and vigorous physical activity and decreasing screen time) [28].

The follow-up pediatrician appointments were brief (~15 min) quarterly visits to review the assessment themes and collaborative goals identified at the initial visit. The pediatricians elicited the perspective of both the child(ren) and parent/guardian regarding progress toward goals and concerns.

### Standard care + enhanced program

The Enhanced Program added components was provided by a bilingual multidisciplinary staff (dietitian, social worker, and fitness instructor). The components included a Skill Building Core (eight weekly sessions) and Post-Core Support (monthly sessions). Development of the Enhanced Program components was guided by evidence-based recommendations and interventions, and clinical experience in the target communities [2, 29, 30].

Motivational enhancement based on Motivation Interviewing (MI) principles was used to engage both parent and child to evoke “their” reasons for changing unhealthy lifestyle behaviors [19]. The staff skill training taught by coauthor (YMR), a member of the international Motivational Interviewing Network of Trainers (MINT), focused on –open-ended questions, affirmations, reflections and summary (ORAS) and using empathic guiding to develop goals collaboratively [31].

The initial Enhanced Program protocol was modified based on feedback obtained from staff and participants during pilot testing. Changes included simplifying print material, reducing the skill-building core from twelve to eight sessions/modules, providing half of the core modules as telephone consultations, limiting self-monitoring to verbal self-reports, and providing up to three module make-up sessions, consistent with methods established in multi-center clinical trial research [32].

#### Skill-building core

The Skill-Building Core sessions, described in Additional file 4: Table S3, alternated between in-person groups and parent/guardian phone consultations. The in-person core group sessions consisted of food preparation or other skill activity for parents/guardians and children, followed by a physical activity session for the children and discussion session for parents/guardians regarding their role in weight management. During the pilot testing, parent/guardians indicated that they preferred to have phone consultations when their children were in school.

#### Post-core support

The monthly Post-Core Support sessions consisted of engagement activities that were designed to provide on-going support to parents/guardians and children during the remainder of the one-year intervention program. A group “meet up” approach was used to provide families with the opportunity to “check in” with Enhanced Program multidisciplinary staff. Themes for post-core sessions included “boot camp” circuit training, holiday themes with active games and outing/field trips to a local park or within the campus grounds.

#### Implementation fidelity procedures

The study manual of procedures for staff training and implementation oversight was used to assure intervention fidelity for Enhanced Program components. Intervention delivery was monitored via intervention logs and session observations. Phone call attempts, completions and duration times were tracked in the trial database. Intervention fidelity was also addressed in bi-weekly intervention staff meetings, using a case discussion approach to address intervention and retention of families in the Enhanced Program. Use of MI in intervention delivery was evaluated by observing, debriefing and discussing intervention sessions using a checklist to assess fidelity to MINT criteria for OARS and empathic guiding to develop goals.

#### Program evaluation by parents/guardians in standard care + enhanced program

Parents/Guardians from families randomized to the Standard Care + Enhanced Program were asked to complete an anonymous program evaluation survey at the end of the 12- month data collection visit. The survey asked the parent/guardian to indicate if program components were helpful using a 4 point scale (definitely yes, maybe yes, maybe no and definitely no). For yes responses, the parents were asked to list places or items that were the most helpful. Short answer response questions were also used to ask the parents/guardians i.e., “What kept you and your family motivated? What kept you and your family from making changes?”

### Study measures, data collection, and assessment blinding

Members of the research team who were involved in obtaining, managing and analyzing primary study outcomes were blinded to the randomized treatment allocation. The pediatricians, who provided the Standard-of-care to both randomization arms, were also blinded to treatment allocation.

Standardized procedures were used to obtain study measurements [33]. Baseline and 12-month post-randomization measures were obtained for all outcomes; BMI was also assessed at 3, 6 and 9 months. Parent/guardian and child health history and demographic information were obtained via questionnaires.

#### Anthropometric measures

Height and weight were measured in light clothing and without shoes. A stadiometer was used to obtain height and a digital scale for weight. Using an inelastic tape, waist circumference was measured at the iliac crest and hip circumference at the point of maximal protrusion of the gluteal muscles in the lateral position, both recorded to the nearest centimeter. Scales and stadiometer were calibrated, and anthropometry tapes were examined for signs of wear on a weekly basis using standardized protocols.

#### Cardiometabolic parameters

Systolic and diastolic blood pressures were measured three times according to traditional pediatric standards using appropriate cuff size with a manual sphygmomanometer after sitting for 2 minutes. Blood specimens were obtained after a minimum of an 8 hour fast. Fasting glucose, triglyceride (TG), total cholesterol (TC), low-density lipoprotein (LDL) cholesterol, and high-density lipoprotein (HDL) cholesterol concentrations were measured spectrophotometrically using a Beckman-Coulter LX-20 auto analyzer (Brea, CA). A glucose load of 1.75 g/kg body weight (Glucola™) was administered for the 2-h Oral Glucose Tolerance Test. The liver enzymes alanine transaminase (ALT) and aspartate aminotransferase (AST) concentrations were measured using an Immulite 2000 analyzer (Bio-DPC; Siemens Medical, Gywneed, UK).

#### Participant compensation

Stipends were provided to parents for quarterly data collection ranging from US $20–50 with the amount increasing from baseline to 12 months. No monetary compensation was provided for attending intervention sessions. Transportation vouchers (metro cards) were provided for all study visits. However, low-cost gifts such as beach balls, yoyos and Frisbees were given to the children in the Enhanced Program attending skill-building core and post-core support sessions as incentives for their attendance.

### Statistical methods

All analyses were performed using SAS version 9.4 (Cary, NC). Continuous scale demographic and metabolic parameters were numerically summarized using mean, standard deviations (median/range) and frequency count and percentage for categorical variables. Comparisons of baseline characteristics between the treatment arms were performed using Student t-test or Wilcoxon rank sum test for continuous variables as appropriate and chi-square test for categorical variables.

#### Power analysis

Initial power analysis focused on the difference between the arms in the decrease BMI Z-score at the end of study from baseline using t-test. In these analyses, it was estimated that 202 participants per arm would be required to achieve 80% power to detect a between-arm difference of 0.07 in a change in BMI-Z score with an alpha set at 0.05. An interim power analysis was conducted approximately 9 months into the study at which point it became apparent that it would be difficult to reach the target sample size.

The final power analysis focused on the rate of BMI-Z score change incorporating all data values rather than the difference in BMI-Z score at the end of the study. Estimates of the rate of BMI-Z score were based on longitudinal random coefficient model. In these analyses, under the intention to treat analysis for 1 month increase in time, the estimated decrease in BMI-Z score for the Enhanced Program was 0.002 (SE: 0.003, *P* = 0.4219), numerically favoring the enhanced program. Based on these results, it was estimated using longitudinal sample size calculations [34, 35] that 1433 participants per arm would provide 80% power to detect an estimate of 0.002 in the final analysis.

An as-treated analysis of Enhanced Program participants with >6 contacts versus Standard Care Alone showed an estimated average decrease in BMI-Z score for the Enhanced Program of 0.008 (SE: 0.005, *P* = 0.0803); this marginally significant finding favored the Enhanced Program. Based on this finding, it was estimated that 192 participants per arm would provide 80% power to detect a difference in slope of 0.008 in the final analysis.

#### BMI Z-score change- intention to treat analysis

The primary outcome of interest, BMI Z-score, was compared between randomization arms throughout follow-up using a generalized linear mixed effects model [36]. The model enables a repeated-measurement analysis with irregular mistimed measurements with serial correlation and missing data, with randomization arm, time and time x randomization arm along with adjusting for other covariates as fixed factors. A random intercept and a random slope model were fitted to represent individual trajectories using restricted maximum likelihood procedure with unstructured random effects covariance structure. This approach models the underlying BMI Z-score trajectory for the individual child at various time points and then compares the average trajectories between treatment arms; missing data were assumed as missing at random.

#### Intervention dose-response analysis

Established methods for intervention dose-response evaluation [37] were used to assess attendance data, and rate of BMI Z-score change within each subgroup using the as-treated principle. The intervention dose for Standard Care Alone was categorized by the number of pediatrician visits. Our analysis categories were 1–2 pediatrician visit encounters, 3 visits, or 4 visits. The intervention dose for the Standard Care + Enhanced Program was categorized by the number of contacts for completing the Skill Building Core. Having >6 contacts for completing the Core was categorized as high dose while ≤6 contacts for completing the Core was categorized as a low dose. Participants in the Standard Care Alone were analyzed as a control arm.

#### Metabolic change

Metabolic parameters evaluated at baseline and 12-months were analyzed using paired t-test or Wilcoxon sign rank test within each arm; the difference between and within arms adjusting for child’s age, gender and race/ethnicity were assessed using a covariance pattern repeated measurement model with unstructured covariance [38]. Household income was not significant and not retained in the final model. Akaike Information Criterion was used in selecting the best model [38].

## Results

At baseline the mean age of the participants (*n* = 360) was 9.3 (±1.7 SD) years; about half were female and about three-quarters self-identified as Hispanic/Latino (Table 1). Almost half of parents/guardians had less than a high-school education, and almost three-quarters reported less than $30,000 annual income. There were no significant differences in baseline characteristics between the randomization arms.

**Table 1:** Baseline participant characteristics by randomization group

Characteristics	Standard Care Alone (n = 182)	Standard Care + Enhanced Program (*n* = 178)	*p*-value
Age in years, mean (SD)	9.3 (1.7)	9.3 (1.7)	0.67
Male, *n (%)*	94 (51.6)	81 (45.5)	0.24
Race/Ethnicity, *n (%)*			0.47
Hispanic/Latino	132 (72.5)	135 (75.8)	
Non-Hispanic Black	36 (19.80)	27 (15.2)	
White, Asian, and others	14 (7.8)	16 (9.0)	
Spanish Spoken in Home, *n (%)*	112 (61.5)	108 (60.7)	0.87
Parent/Guardian highest education			0.12
level, *n (%)*	79 (43.4)	96 (53.9)	
< High School	55 (30.2)	41 (23.0)	
High School or GED	48 (26.4)	41 (23.0)	
> High School			
Household income, *n (%)*			0.33
≥ $30,000	14 (7.7)	22 (12.4)	
< $30,000	133 (73.1)	125 (70.2)	
Unknown	35 (19.2)	31 (17.4)	
BMI Z-score, mean (SD)	2.02 (0.39)	1.95 (0.42)	0.12

*BMI*, Body Mass Index

*GED*, General Equivalency Diploma

### BMI Z-score change

**Fig. 2:**
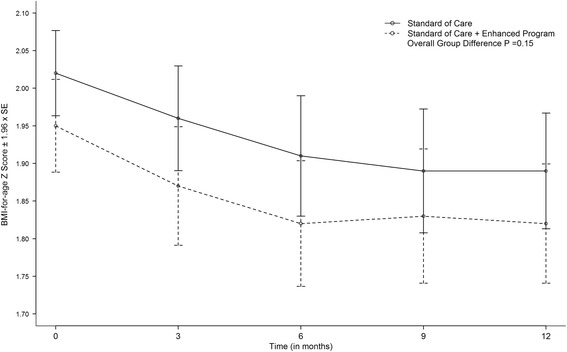
Change in body mass index Z-score by randomization group. Change in body mass index (BMI) Z-score by randomization group from baseline to 12-months (*n* = 182 in Standard Care Alone (control) intervention; *n* = 178 in Standard Care + Enhanced Program (experimental) intervention. The rate of change in the mean BMI Z-score decreased 0.12 (SE: 0.03) units in the control intervention vs. 0.15 (SE: 0.03) in the experimental intervention (<0.01) per 12-months for both interventions with no significant difference observed between the groups (*P* = 0.15)

During the 12-month intervention period, the trajectory of change in BMI Z-scores was similar in both intervention arms (*p* = 0.15; Fig. 2). The estimated regression coefficients for BMI Z-score from linear mixed effects model are provided in Additional file 5: Table S4. The rate of change in the mean BMI Z-score decreased 0.12 (*p* < 0.01) units within the Standard Care Alone arm and 0.15 unit in the Standard Care + Enhanced Program arm (*P* < 0.01 for both arms). The rate of change (slopes) was similar between the two arms (0.03; *p* = 0.42). Older children had a greater decline in BMI Z-score than younger children (beta −0.04 units for each additional year of age; P = <0.01). Girls exhibited a greater decline in BMI Z-score than boys, (β = 0.09 *P* = 0.03).

### Intervention contact response

The Standard Care Alone arm had a median of three visits during the 12-month period (range: 1–4). A subgroup analysis within the Standard Care Alone arm suggested no significant effect of number of contacts (≤ 2, 3, 4) on the rate of change (slope) of BMI Z-score (*p* = 0.27). The Standard Care + Enhanced Program arm had a median of six contacts with Enhanced Program multidisciplinary staff and completed 5.7 ± 2.2 (mean ± SD) Skill Building Core modules. Eighty-five percent of the families completed all eight core modules during 5.2 ± 1.5 (mean ± SD) contacts. A sensitivity analysis comparing the Standard Care Alone (control arm) to the low dose intervention (≤ 6 Skill Building Core contacts) indicated there was no significant difference in the rate of change in BMI Z-score (0.001 [SE:0.003]). Although the overall difference in the rate of change among the arms was not significantly different (*p* = 0.20), the 21.9% of children (*n* = 39) from high contact families (> 6 contacts) had a larger decrease in BMI Z-score (of approximately 0.01 unit [SE:0.005] BMI Z-score) than the Standard Care Alone (control), and this difference approached statistical significance (*p* = 0.08).

### Metabolic parameter changes

The Standard Care + Enhanced Program arm had significant improvements in HDL cholesterol (*P* = 0.02), LDL cholesterol (*P* = 0.01), AST (*P* = 0.01), and ALT (*P* = 0.01) concentrations. In contrast, the Standard Care Alone arm had a significant improvement in AST (*P* = 0.01) levels but an increase in triglyceride (*P* = 0.01) concentrations. Compared to the Standard Care Alone, the Standard Care + Enhanced Program achieved significantly greater improvements in total cholesterol (*P* = 0.05), LDL cholesterol (*P* = 0.04), AST (*P* = 0.02), and ALT (*P* = 0.03) concentrations (Table 2).

**Table 3:** Evaluation of Components by Parents/Guardians in Standard Care + Enhanced Program ^a^ (n = 114)

Did __ help your child/children [change (eating or physical activity) habits)?	Definitely Yes n (%)	Maybe Yes n (%)	Maybe No n (%)	Definitely No n (%)	Not Applicable n (%)
Receiving the newsletter	89 (78.1)	13 (11.4)	6 (5.3)	3 (2.6)	3 (2.6)
Talking to (study) doctors	95 (83.3)	13 (11.4)	2 (1.8)	3 (2.6)	1 (0.9)
Meeting with (via referral) social worker or dietitian	39 (34.2)	15 (13.2)	1 (0.9)	5 (4.4)	54 (47.4)
Talking about “my Plate Planner”^b^ (control portion size)	87 (77.7)	18 (16.1)	4 (3.6)	2 (1.8)	1 (0.9)
Talking about “my Plate Planner” (chose food)	84 (75.0)	19 (17.0)	6 (5.4)	2 (1.8)	1 (0.9)
Talking about the nutrition label	91 (81.3)	14 (12.5)	4 (3.6)	2 (1.8)	1 (0.9)
Talking about smart super market shopping	86 (77.5)	17 (15.3)	2 (1.9)	2 (1.9)	4 (3.6)
Learning about places to buy healthy food in your neighborhood	69 (61.6)	19 (17.0)	9 (8.0)	8 (7.1)	7 (6.2)
*List most helpful* ^c^ Local farmers’ market, local supermarket					
Learning about Website	17 (15.2)	4 (3.6)	19 (17.0)	35 (31.3)	37 (33.0)
*List most helpful* Websites with nutrition information, recipes					
(Study) Recipes	45 (40.2)	17 (15.2)	15 (13.4)	14 (12.5)	21 (18.8)
*List most helpful* Quesadilla, taco recipes					
Completing fitness testing	59 (52.7)	20 (17.9)	14 (12.5)	9 (8.0)	10 (8.9)
*What were the most helpful activities?*	Exercise classes, walking, seeing food portion sizes.				
*What kept you and your family motivated?*	Coming to see the staff, improving child(ren)‘s health, having family support				
*What kept you and your family from making changes?*	Lack of money e.g., “healthy food is expensive, finances, Work or school schedules” e.g., “her school schedule made it difficult to come.” Lack of family social support e.g., “grandmother gives her everything she wants to eat.”				

^a^Anonymous end-of-study survey completed by parents/guardians randomized to Standard Care + Enhanced Program

^b^“my Plate Planner” is an educational tool from the NYC Department of Health and Mental Hygiene https://www1.nyc.gov/assets/doh/downloads/pdf/csi/obesity-plate-planner-13.pdf

^c^Italicized questions were designed to elicit short answer responses i.e., listing of most helpful items in the category or what kept the family motivated or kept the family from making changes

### Program evaluation by parents/guardians in standard care + enhanced program

The anonymous Program Evaluation survey (See Table 3) was completed by 64% (*n* = 114) of parents/guardians of children from families randomized to the Standard Care + Enhanced Program. Over half of the parents/guardians gave the highest rating (definitely yes) to eight of the eleven questions about program components i.e., “Did __ help your child/children change (eating or physical activity) habit?” Almost half (47.4%) indicated that meeting (via physician referral) with the social worker or dietitian was not applicable. About one-third indicated that learning about websites was not applicable, and less than 20% responded that learning about websites was definitely or maybe helpful in changing eating or physical activity habits. In response to the short answer questions, parents/guardians listed exercise classes, walking and food portion sizes as helpful most frequently. Reported barriers (what kept the family from making change) included lack of money, scheduling problems and lack of family support. Parents/guardians indicated that meeting with staff, perceiving an improvement in child’s health and family support, kept them motivated.

**Table 2:** Cardiometabolic Risk Parameters at Baseline and 12 Months by Randomization Group

Cardiometabolic Risk Parameters	Standard-of-Care Alone (n = 182)			Standard-of-Care + Enhanced Program (n = 178)			Difference in mean slope /12 months^a^	Difference in mean slope^a^	Overall group difference^a^
	Baseline	12 Months	*p*-value^b^	Baseline	12 Months	*p*-value^b^	Experimental - Control ±SE	*p*-value	*p*-value
Systolic blood pressure, mean (SD), mmHg	106.6 (10.2)	107.1 (12.9)	0.37	106.7 (11.1)	107.9 (12.3)	0.13	0.59 ± 1.36	0.67	0.87
Diastolic blood pressure, mean (SD), mmHg	58.7 (5.5)	58.8 (5.9)	0.94	58.1 (5.7)	58.0 (5.6)	0.87	−0.05 ± 0.74	0.94	0.38
Fasting Glucose, mean (SD), mmol/L	4.7 (0. 6)	4.8 (0.4)	0.19	4.7 (0.5)	4.8 (0.4)	0.13	−0.02 ± 0.06	0.70	0.65
2 Hour Glucose, mean (SD), mmol/L	5.4 (1.0)	5.66 (1.1)	0.11	5.4 (1.0)	5.4 (1.0)	0.74	−0.14 ± 0.13	0.25	0.18
Total Cholesterol, mean (SD), mmol/L	4.09 (0.77)	4.15 (0.75)	0.60	3.98 (0.69)	3.87 (0.69)	0.19	−0.1 ± 0.06	0.10	0.05
HDL Cholesterol, mean (SD), mmol/L	1.19 (0.24)	1.24 (0.27)	0.28	1.19 (0.26)	1.23 (0.30)	0.02	0.01 ± 0.02	0.48	0.67
LDL Cholesterol, mean (SD), mmol/L	2.46 (0.59)	2.41 (0.61)	0.07	2.35 (0.60)	2.18 (0.56)	0.01	−07 ± 0.05	0.11	0.04
Triglycerides, mean (SD), mmol/L	1.02 (0.71)	1.11 (0.61)	0.01	0.94 (0.49)	1.01 (0.56)	0.09	−0.06 ± 0.06	0.34	0.08
AST, mean (SD), ukat/L	0.50 (0.12)	0.45 (0.23)	0.01 ^c^	0.53 (0.19)	0.40 (0.19)	0.01^c^	−0.08 ± 0.03	0.01	0.02
ALT, mean (SD), ukat/L	0.41 (0.19)	0.45 (0.23)	0.15	0.45 (0.37)	0.40 (0.19)	0.01 ^c^	−0.08 ± 0.03	0.01	0.03

^a^Based on covariance pattern model and adjusted for child’s age, sex and ethnicity

^b^Paired t-test

^c^Wilcoxon sign rank test

*HDL*, High-density lipoprotein*, LDL*, Low-density lipoprotein*, ALT*, alanine transaminase*, AST,* aspartate aminotransferase

## Discussion

The main study finding was that children in both the Standard Care Alone and Standard Care + Enhanced Program arms achieved a significant decrease in BMI Z-score during the 12-month intervention period in response to an embedding weight management program consistent with the American Academy of Pediatrics overweight/obesity recommendations. The magnitude of BMI Z-score change is comparable to a family weight management program conducted in a more affluent, largely Caucasian study population [39] and better than a family-based intervention program conducted in a low-resource community setting, despite significant improvements in self-reported behavioral changes in the latter setting [14].

The addition of an Enhanced Program (skill-building core and follow-up post-core support) to the Standard Care intervention did not result in the hypothesized significantly greater decrease in BMI Z-score after the 12 month intervention period. Our study sample size was calculated to evaluate the potential superiority of adding the enhanced program rather than to evaluate intervention benefit equivalence. We selected our control intervention, which systematically implemented care standards in scheduled visits rather than using a minimal potentially sub-standard control, on the basis of ethical considerations.

Both randomization groups achieved significant improvements in BMI Z-score with no significant difference between the randomization groups. However, children from high contact families (> 6 contacts) for the Enhanced Program tended to have a larger decrease in BMI Z-score than the Standard Care Alone (control) (*p* = 0.08). While 85% of families completed all of the 8 modules, only 22% of families had >6 contacts. The trend for greater BMI Z-score improvement is consistent with Janicke et al. meta-analysis which found that increasing the number of contacts was a strong predictor of BMI Z-score improvement [6].

The Standard Care + Enhanced Program achieved significantly greater improvements in metabolic parameters than Standard Care Alone. The unanticipated rise in triglyceride concentrations in Standard Care Alone arm was likely related to hormonal changes associated with pubertal development, [40] rather than any adverse effect of the intervention. Although there were no significant baseline differences between the randomization arms, the Standard Care Alone arm tended to have a higher proportion of boys. The predictors of BMI Z-score improvement included being a girl and older. Predictors of metabolic changes will be addressed in a future manuscript considering the sexual maturation and the earlier age of pubertal development in girls than boys as potential contributors to metabolic improvement in relations to BMI Z-score improvement.

The program evaluation suggests that the parents/guardians found most of the intervention components helped their child/children change eating or physical activity habits. Components that were less frequently used included meeting (via physician referral) with a social worker or dietitian and learning about websites. Parents/guardians reported that barriers to making positive changes in diet quality and physical activity included time constraints, higher cost of healthy foods, transportation difficulties, and inadequate family support. Nearly 40% of the parents/guardians indicated that their family had food insecurity (worry about running out of money to buy food), which was associated with a greater likelihood of pressuring their child(ren) to eat [18].

## Study limitations

Generalizability of our findings may be limited by the demographic characteristics of study population, institutional characteristics, enrollment of about half of eligible families, and reliance on make-up sessions. However, the need to leverage publically funded health-care resources to address the burden of excess body weight in ethnic minority and low-income youth is universal and should be explored further [8].

The study may have been under powered, potentially because the effect of the Standard Care quarterly consults provided by the experienced and highly skilled study pediatricians was greater than anticipated. Although we did not have a minimal intervention control arm, our study demonstrated the benefit of primary care pediatricians systematically addressing weight management care recommendations on a quarterly basis. Achieving frequent intervention contact appears to be particularly challenging. Despite making changes to accommodate the needs of the parents/guardians, delivering the intervention in eight contact sessions proved difficult. An average of one-and-a-half modules had to be addressed during each Skill Building Core contact session. Prior research has suggested frequency of weight management intervention contacts is a key predictor of change in BMI Z-score [6]. Digital technology has the potential for increasing contact frequency, but less than 20% of the parents/guardians indicated learning about websites was helpful in changing eating or physical activity habits. Text messaging may be more acceptable as a venue for maintaining frequent contact in weight management programs for low-income families with Spanish-speaking parents [41].

Self-reported data may be biased due to the effects of socially desirable responses. The highly favorable program evaluation limits the lessons learned and insights for improving weight management services, but the low usage and potentially low interest in using websites posed issues that need further refinement. Our program evaluation survey did not assess the perceived helpfulness of intervention delivery by telephone or the potential use of other digital technology or social media to promote healthier eating and physical activity. Input from youth with a BMI ≥ 85th percentile and their parents/guardians could provide valuable insights with regard to using digital platform and social media in weight management. Stakeholder feedback needs to address the facilitators and barriers to healthier eating and physical activity. The mandated annual Community Health Needs Assessment provides a venue for eliciting the opinions of parents/guardians about weight management services delivered by telephone calls, texting, or use of other digital technology [42, 43].

### Study strengths

Study strengths include collaborative goal-setting to empower parents/guardians and children to make informed lifestyle choices [44, 45], focusing on an underserved high risk largely Hispanic population, using electronic health records to identify eligible children, and retaining >80% of participants. During enrollment some parents declined study enrollment due to time and other commitments that prevented them from participating in a more intensive weight management program. Therefore, increasing the internal validity (80% retention) based on explanatory trial criteria may have reduced the external validity (generalization) of findings based on pragmatic trial criteria [46].

## Conclusions

Our findings suggest that safety-net care settings can address weight management standards in quarterly family consults to achieve a modest BMI Z-score improvement in children whose BMI is ≥ the 85th percentile. Adding an enhanced program may achieve greater metabolic improvement that may be independent of weight change since the Enhanced Program did not achieve a greater reduction in BMI Z-score. Pediatric overweight and obesity should be managed as a chronic condition in public hospital safety-net ambulatory care programs and other primary care settings. Including weight-management services in publically funded primary care programs could address the needs of low-income families to help prevent as well as treat excess weight gain.

### Clinical implications

Children who are ≥85th BMI percentile for age and sex can be readily identified in public hospital setting using EHR data, and their families can be recruited to participate in a weight management program in collaboration with primary care providers. Embedding family weight management services within pediatric primary care practices makes such services more readily available without complex referral procedures, which can be a barrier for families with few resources and may not speak the dominant culture language. However, hospital-based weight management programs may need greater community engagement to address barriers to achieving healthy eating and physical activity habits in low resource communities when children from immigrant families are at risk for becoming obese. Questions about weight management program delivery need to be incorporated into hospital community health needs assessment. The Affordable Care Act mandates that not-for-profit hospitals in the United States update assessments and service plans annually, and hospitals and other health service providers need to address the more complex issues that contribute to low resource community health disparities including obesity [42, 43, 47].

Future research should engage non-English speaking parents/guardians as stakeholders in developing, implementing and evaluating weight management programming for immigrant families whose child/children are at risk for or have gained excess body weight. Their input may provide valuable insights for how to increase contact frequency, such as offering healthy lifestyle programs in convenient locations to minimize travel time and considering digital options that are more likely to engage families who are least likely to participate.

## Additional files

Additional file 1: Table S1. Available Resources and Intervention Components by Randomization Group. (DOCX 17 kb)

Additional file 2: CONSORT 2010 checklist of information to include when reporting a randomised trial. (DOC 219 kb)

Additional file 3: Table S2. Tools and Handouts Used by Pediatricians for Standard of Care Quarterly Consults. (DOCX 20 kb)

Additional file 4: Table S3. Skill-Building Core Sessions (Enhanced Program). (DOCX 22 kb)

Additional file 5: Table S4. Intention-To-Treat Analysis BMI Z-Score Change by Time and Intervention (*n* = 360). (DOCX 15 kb)
