# Supplementary material for: Embedding weight management into safety-net pediatric primary care: randomized controlled trial
Source: Int J Behav Nutr Phys Act. 2018 Jan 22;15:12. doi: 10.1186/s12966-017-0639-z (PMC5778780; doi:10.1186/s12966-017-0639-z)
Supplement: Supplementary file 1 — Available Resources and Intervention Components by Randomization Group. (DOCX 17 kb) [file 12966_2017_639_MOESM1_ESM.docx]

**Supplemental Table 1. Available Resources and Intervention Components by Randomization Group**

| **Resources/ Component** | **Standard-of-Care Alone** | **Standard-of-Care + Enhanced Program** |
| --- | --- | --- |
| Farmer’s Market on Jacobi Medical Center Campus  *Available June – November* | - Participants were provided a Health Bucks card (value $2 American) and additional cards were available using the Supplemental Nutrition Assistance Plan [electronic benefit transfer](javascript:searchFor('electronic%20benefit%20transfer%20ebt')) card. | Same as Standard-of- Care Alone |
| Gym on Jacobi Medical Center Campus | Not Used | Used for:   - Skill Building Core in-person physical activity sessions (4 sessions) - Post-Core Support Session (used for selected sessions e.g. boot camp) |
| WAVE - Get Healthy Game Card^12-14^  *Available in English/Spanish* | Used to engage children in self-assessment and setting goals | Same as Standard-of Care Alone |
| Standard-of-Care Pediatrician Visits (4 Visits)  *Provided by embedded bilingual study pediatricians in the designated weight management clinical sessions.* | Initial Visit (assessment/collaborative goal setting)  35-item Pediatric Symptom Checklist (PSC)^15-18^  5-item Habits Questionnaire^19^  Follow-Up Visits (quarterly follow-up visits to address themes/goals from initial visit) | Same as Standard-of-Care Alone |
| Newsletter (Monthly)  *Available in English/Spanish* | Recipes and Weight Management Behavior Tips | Same as Standard-of-Care Alone |
| Skill-building Core (8 sessions)  *Provided by bilingual multidisciplinary staff.* | Not included | See Supplemental Table 2 for curriculum.  Alternated between in-person groups (child(ren) + parent/guardian) and telephone consults (parent/guardian alone) |
| Post-Core Support Sessions (monthly)  *Provided by bilingual multidisciplinary staff.* | Not included | Provided monthly |
