# Supplementary material for: Embedding weight management into safety-net pediatric primary care: randomized controlled trial
Source: Int J Behav Nutr Phys Act. 2018 Jan 22;15:12. doi: 10.1186/s12966-017-0639-z (PMC5778780; doi:10.1186/s12966-017-0639-z)
Supplement: Supplementary file 3 — Tools and Handouts Used by Pediatricians for Standard of Care Quarterly Consults. (DOCX 20 kb) [file 12966_2017_639_MOESM3_ESM.docx]

**Supplemental Table 2. Tools and Handouts Used by Pediatricians for Standard of Care Quarterly Consults**

| **Tools and Handouts-Available from the New York City Department of Health and Mental Hygiene** |
| --- |
| http://www1.nyc.gov/site/doh/health/health-topics/obesity.page |
| Pouring on the weight  Make NY Your Gym  Eating out  Plate planner |
| https://www1.nyc.gov/assets/doh/downloads/pdf/csi/csi-obesity-in-children-folder.pdf (Obesity in Children Action Kit) |
| [My Health Goals Pad](http://www.nyc.gov/html/doh/downloads/pdf/csi/csi-my-health-goals-pad.pdf) [My Child’s Plate Planner](http://www.nyc.gov/html/doh/downloads/pdf/csi/csi-my-child-plate-planner.pdf) [What’s a Healthy Portion Size? Flipchart](http://www.nyc.gov/html/doh/downloads/pdf/csi/csi-portion-size-flipchart.pdf)  [You Have the Power! You can Make Healthy Choices. Goal Setting Poster](http://www.nyc.gov/html/doh/downloads/pdf/csi/csi-you-have-the-power.pdf)  Patient Education  Helping Children Reach a Healthy Weight Booklet |
| https://www1.nyc.gov/assets/doh/downloads/pdf/public/dohmhnews9-01.pdf |
| Health Bulletin 9: No. 1 (series No. 76) Good Fat/Bad Fat |
