# Supplementary material for: Embedding weight management into safety-net pediatric primary care: randomized controlled trial
Source: Int J Behav Nutr Phys Act. 2018 Jan 22;15:12. doi: 10.1186/s12966-017-0639-z (PMC5778780; doi:10.1186/s12966-017-0639-z)
Supplement: Supplementary file 4 — Skill-Building Core Sessions (Enhanced Program). (DOCX 22 kb) [file 12966_2017_639_MOESM4_ESM.docx]

**Supplemental Table 3. Skill-Building Core Sessions (Enhanced Program)**

| **Core Session Title/Format** | **Learning Objectives** |
| --- | --- |
| 1. Integrating the Family Weight Program into Your Life: WELCOME!   (In-Person Group - Child(ren) and Parent/Guardian)^a^ | Learn about the program goals  Strengthen family bonds  Discuss group rules/etiquette.  Review goals set randomization  Parent/guardian - learn how to: role model and support child(ren) |
| 1. Understanding the Food Groups   (Phone - Parent/Guardian) ^b^ | Identify food groups  Practice planning meals  Use plate to address portion sizes |
| 1. Be a Label Detective   (In-Person Group - Child(ren) and Parent/Guardian) ^a^ | Learn to read a nutrition facts label  Compare food choices when shopping and at vending machines |
| 1. Smart Shopping Strategies & Limiting Sugary Drinks   (Phone - Parent/Guardian) ^b^ | Evaluate supermarket layout  Develop plan for smart supermarket shopping  Identify beverages with hidden sugar  Estimate the total number of teaspoons of sugar consumed over a day |
| 1. If Five Fruits/Vegetables a Day is Fine, Could Nine-a-Day be Divine?   (In-Person Group - Child(ren) and Parent/Guardian) ^a^ | Learn about the taste variety and benefits of eating a variety of fruits and vegetables  Use the concept of eating a variety of fruits/vegetables from the color spectrum |
| 1. Managing Criticism & Stress and Enhancing Self- Esteem   (Phone - Parent/Guardian) ^b^ | Identify sources of stress  Brainstorm how to deal with stress  Promote self-esteem and positive responses to stress in child(ren) |
| 1. How to Increase Physical Activity and Reduce Screen Time   (In-Person Group - Child(ren) and Parent/Guardian) ^a^ | Identify alternatives to watching TV/screen time  Identify ways child(ren) can be more physically active  Develop plan to be active together as a family |
| 1. Limit Fast Foods & Junk Foods and Recognize Advertisement Tricks   (Phone - Parent/Guardian) ^b^ | Compare the nutrition facts label of fast food menu items  Identify trends in sample advertisements and discuss advertising “tricks” |

a All in-person group modules included a 45 minute breakout to provide physical activity session for the children and problem-solving discussion for the parents/guardians. An abbreviated version of each physical activity session was offered to the parents/guardians after they completed their discussion session.

b All phone modules included 20-30 minutes of included nutrition content (noted above) and addressed potential barriers to being physically active at home (e.g. physical space, apartment setting and/or setting with limited or no outdoor space, noise within space.
