# Supplementary material for: Embedding weight management into safety-net pediatric primary care: randomized controlled trial
Source: Int J Behav Nutr Phys Act. 2018 Jan 22;15:12. doi: 10.1186/s12966-017-0639-z (PMC5778780; doi:10.1186/s12966-017-0639-z)
Supplement: Supplementary file 5 — Intention-To-Treat Analysis BMI Z-Score Change by Time and Intervention (n = 360). (DOCX 15 kb) [file 12966_2017_639_MOESM5_ESM.docx]

| **Supplemental Table 4. Intention-To-Treat Analysis BMI Z-Score Change by Time and Intervention (n=360)** | | | |
| --- | --- | --- | --- |
| Variables | β | SE | p-value |
| Intercept | 2.60 | 0.13 | <0.001 |
| Time (Months) | -0.01 | 0.002 | <0.001 |
| Study Arm: Standard-of-Care + Enhanced Program | -0.07 | 0.04 | 0.10 |
| Time x Study Arm: Standard-of-Care + Enhanced Program | -0.002 | 0.002 | 0.42 |
| Child Age (Years) | -0.04 | 0.01 | 0.001 |
| Gender : Female | -0.09 | 0.043 | 0.03 |
| Ethnicity/Race: Hispanic / Latino  White, Asian, and others  Non-Hispanic Black | -0.16  -0.19  Reference | 0.06  0.09  Reference | 0.01  0.03  Reference |

*Results from linear mixed effects model accounting for random intercept and slope using estimated regression coefficients (fixed effects), standard error and P value for BMI Z-score.*
